# Cost-effectiveness of nivolumab plus ipilimumab as first-line treatment for American patients with unresectable malignant pleural mesothelioma

**DOI:** 10.3389/fpubh.2022.947375

**Published:** 2022-07-22

**Authors:** Zhuo-miao Ye, Zi-Qing Tang, Zhe Xu, Qin Zhou, Huan Li

**Affiliations:** ^1^Department of Oncology, Xiangya Hospital, Central South University, Changsha, China; ^2^Department of Pharmacy, Xiangya Hospital, Central South University, Changsha, China

**Keywords:** cost-effectiveness, nivolumab, ipilimumab, malignant pleural mesothelioma, Markov model

## Abstract

**Background:**

The treatment paradigm of unresectable malignant pleural mesothelioma (MPM) has changed in recent years. Checkmate 743 demonstrate that nivolumab plus ipilimumab showed good clinical benefits compared with chemotherapy in the treatment of MPM. The study is aim to evaluate the cost-effectiveness of Nivolumab plus ipilimumab vs. platinum plus chemotherapy for the first-line treatment of unresectable MPM.

**Methods:**

A Markov model was developed to compare the cost and quality-adjusted life-year (QALY) of nivolumab plus ipilimumab and chemotherapy over a 10-year time horizon. Clinical efficacy and safety data were extracted from the CheckMate 743 trials. Health state utilities were obtained from published literature. Costs were collected from an US payer perspective. One-way and probabilistic sensitivity analyses were conducted to explore the impact of uncertainties on the cost-effectiveness's results.

**Results:**

In the base case analysis, the incremental healthcare costs and QALYs for Nivolumab plus Ipilimumab vs. chemotherapy are $196,604.22 and 0.53, respectively, resulting an incremental cost-effectiveness ratio (ICER) of $372,414.28/QALYs for the model cohort of patients with locally advanced or metastatic MPM. However, Probabilistic sensitivity analysis showed that there was no probability that Nivolumab plus ipilimumab was cost-effective within the fluctuation range of other model parameters in first-line in unresectable MPM. The results of one-way sensitivity analysis showed that the cost of Nivolumab was the most sensitive parameter.

**Conclusions:**

The ICER of Nivolumab plus ipilimumab is above the theoretical willingness-to-pay threshold in the U.S, which suggests that first-line nivolumab plus ipilimumab for unresectable MPM may be not a cost-effective choice.

## Introduction

Malignant pleural mesothelioma (MPM) is an uncommon but fatal malignancy with high aggressiveness. The number of new cases of MPM globally is 30,870 and the number of deaths is 26,278 according to the statistics in 2020 (1). Its pathogenesis is strongly associated with prior exposure to asbestos, and that caused high incidence of MPM in developed countries (2). Despite the widespread prevention, the incidence and mortality of MPM is still increasing and will reach its maximum after about 20 years, especially in developing countries, because of the long latency of asbestos exposure (3). The prognosis of malignant mesothelioma is poor. The survival rate at 5 years is <10% and the median overall survival of the diagnosed patients is 7.9 months (4, 5).

The great majority of patients of MPM are not diagnosed until advanced stage, so only a minority of patients can be treated with surgery. For a long time in the past, the only first-line treatment that FDA (U.S. Food and Drug Administration) approved for unresectable MPM is a platinum-based chemotherapy combined with pemetrexed. However, the efficacy is modest (6). A better treatment is urgently needed. Biological targeted therapy for MPM is not recommended because of the lack of known oncogenic driver alterations (7). Clinical studies of MPM immunotherapy has underwent a transition from relapse to second-line, monotherapy to combination, and now seemed promising in some phase II clinical studies (8). Based on these trials, the NCCN guidelines (2018) recommend nivolumab ± ipilimumab or pembrolizumab as subsequent systemic therapy (9).

The phase III randomized trial Checkmate 743 (NCT02899299), in which compare the curative effect and safety of cisplatin (or carboplatin)-pemetrexed and that of nivolumab-ipilimumab as first-line treatment, also show a promising result. It demonstrated that nivolumab-ipilimumab prolonged overall survival in comparison with the chemotherapy group [median 18.1 vs. 14.1 months; hazard ratio (HR), 0.74 (96.6% CI 0.60–0.91); *p* = 0.0020]. Two-year overall survival rates were 41% (95% CI 35.1–46.5) vs. 27% (21.9–32.4). In addition, among all treated patients, 91 (30%, nivolumab plus ipilimumab) and 91 (32%, chemotherapy) patients had grade 3–4 treatment-related adverse events. The overall incidence of treatment-related adverse events adjusted for exposure in nivolumab-ipilimumab group is lower than that in chemotherapy group (10). According to the results, nivolumab plus ipilimumab has been approved by the FDA to treat the unresectable MPM as a first-line therapy in October 2020 (11).

Despite the efficiency and safety of nivolumab-ipilimumab combination therapy for unresectable MPM, what we need to evaluate further is the cost-effectiveness of these drugs when considering both drugs have high unit prices which may add heavy financial burden to the patients (12). The objective of this study is to evaluate the cost-effectiveness of nivolumab plus ipilimumab vs. chemotherapy as the first-line treatment for unresectable MPM from the US payer perspective.

## Materials and Methods

### Population

CheckMate 743 is a randomized phase 3 trial involving 713 patients from November 19, 2016 to April 28, 2018 in 21 countries. Among enrolled patients, 605 were randomized to receive nivolumab plus ipilimumab (*n* = 303) or chemotherapy (*n* = 302). Median age of participants was 69 years (IQR 64–75) and ECOG was 0–1. The median follow-up was 29·7 months (IQR 26.7–32.9). Overall survival [18.1 months (95% CI 16.8–21.4)] was significantly longer in the nivolumab plus ipilimumab group than in the chemotherapy group [14.1 months (95% CI 12.4–16.2)]. Our data were based on clinical characteristics of Checkmate 743 subjects aged 18 years or older with unresectable MPM, regardless of PD-L1 expression.

In the total number of people in this trial, 303 participants were in the experimental group (Nivolumab plus ipilimumab) and 302 in the control group (chemotherapy). We conducted a cost-effectiveness analysis for the Nivolumab plus ipilimumab group and chemotherapy group to provide a foundation for their different treatment. The research methods refer to the consolidated health economic evaluation reporting standards (CHEERS) (see Supplementary Material S1).

### The model's structure

The study used TreeAge Software 2021 (TreeAge Software, Inc., Williamstown, Massachusetts) to program a multi-state Markov model. The purpose was to evaluate the cost-effectiveness of Nivolumab plus ipilimumab and chemotherapy in patients with unresectable malignant pleural mesothelioma. The multiple health states include PFS, progressive disease state (PD) and death. Assuming that patients in a certain state only make one state transition in a cycle, once the patients are in the PD state, they cannot return to the PFS state. Similarly, the patients in the dead state cannot transition to other states. The specific transition relationships are shown in Supplementary Figure S1. We assumed that all the patients were in a PFS healthy state at the model's initial stage. The patients were treated with Nivolumab plus ipilimumab or chemotherapy according to their groupings. When the disease progresses, the follow-up treatment plan in the CheckMate 743 clinical trial is used for treatment until the patient's death.

We developed a Markov model to simulate the patient's entire life course and evaluate the cost and effectiveness of first-line therapy for patients with unresectable malignant pleural mesothelioma. In the CheckMate 743 clinical trial, the median survival time of the experimental group was 18.1 months, the control group was 14.1 months. The effect of immunotherapy has a delayed effect and may continue to work beyond the treatment period. It should be analyzed from long-term data to avoid inaccuracy and uncertainty of results (13, 14). As the result of that, with reference to the dosing cycle of the CheckMate 743 clinical trial, we set the cycle of the Markov model to 3 weeks and the time range was 10 years. Approximately 99% of patients were in the absorption state (15). A half-circle correction was conducted to simulate the transfer process more accurately. Simultaneous simulation analysis of the cost and utility is performed to estimate the cumulative total cost and health outcomes within the cohort's time frame (16, 17). The research was based on the American payers' perspectives, with a 3% discount on costs and utilities (18). According to the World Health Organization, ICER is acceptable when it is below three times GDP per capita (19). This study will use three times of the United States's triple GDP per capita in 2021 is $69,231 as the threshold (World Economic Outlook Database, April 2022, https://www.imf.org/en/Publications/WEO/weo-database/2022/April). The WTP is assumed to be $207,659. The research indicators include the costs, life-years (LYs), quality-adjusted life-years (QALYs), and the incremental cost-effectiveness ratios (ICERs).

### The model's survival and progression risk estimates

The original data for constructing the model were obtained from the CheckMate 743 clinical trial. When some data were unavailable, we referred to the related published literature. The GetData Graph Digitiser (version 2.26; http://getdata-graph-digitizer.com/download.php) was used to extract the Kaplan–Meier curve's data of the PFS and OS in the Nivolumab plus ipilimumab group and the chemotherapy group. We also referred to the algorithm of Guyot et al. who refers to the pseudo-individual patient's data reconstructed by R software (version 4.1.0; https://www.r-project.org/) (20). This was combined with the Akaike Information Criterion (AIC) and the Bayesian Information Criterion (BIC) to select the Log-logistic, Weibull and Log-norm distribution that fitted the survival curve for Nivolumab plus ipilimumab and chemotherapy, respectively, after the reconstruction (21). The distribution has a higher flexibility and estimated distributions (22, 23). Details of model extrapolation are shown in Supplementary Table S1.

### The utility and cost estimates

During the follow-up, the CheckMate 743 trial used the Mesothelioma LungCancer Symptom Scale (LCSS-Meso) to compare quality of life after nivolumab plus ipilimumab vs. chemotherapy as the first-line treatment for unresectable malignant pleural mesothelioma. The average health utility (0.65 for PFS and 0.47 for PD) of the patients with unresectable malignant pleural mesothelioma in the PFS and PD was obtained by the published values for non-small cell cancer (NSCLC) (24), because there is no previously published study mentioning precise utility scores. The top three incidence adverse events (AEs) with grade 3 or above were selected in Nivolumab plus ipilimumab [nivolumab (3 mg/kg intravenously once every 2 weeks) plus ipilimumab (1 mg/kg intravenously once every 6 weeks)] and platinum plus pemetrexed chemotherapy [pemetrexed (500 mg/m^2^ intravenously) plus cisplatin (75 mg/m^2^ intravenously) or carboplatin (area under the concentration-time curve 5 mg/mL per min intravenously)] to be considered to evaluate the loss of the health utility caused by the three to five adverse events (AEs) for simplifying the calculation. The top three incidence adverse events (AEs) with grade 3 or above in Nivolumab plus ipilimumab and chemotherapy are Diarrhea (0.3%), Increased lipase (0.5%), Increased amylase (0.3%) and Anemia (36%), Nausea (36%), Decreased appetite (18%), respectively.

The costs are reported in 2021 US dollars (US $1.0 = CNY 6.4). Only the direct costs of the medical expenses were considered. This included the cost of the drugs, subsequent treatment costs, management costs, follow-up costs, laboratory examination costs, and the major adverse reactions with grade 3 or above had the top three incidence rates according to CheckMate 743 trial.

The estimated cost of each drug during the set period is listed in Table 1. The probability that different treatment groups intend to receive different follow-up treatment and the treatment mode of specific subsequent therapies [systemic therapy other than PD-(L)1 inhibitors, local regional therapy, radiation therapy, surgery, PD-(L)1 inhibitors] are derived from CheckMate 743 trial (10, 24–33).

**Table 1:** Baseline value.

**Variable**	**Baseline value**	**Range**			**Distribution**	**References**
		**Minimum**	**Maximum**			
Weilbull OS survival model in Nivolumab plus ipilimumab	Shape = 1.19536, scale = 25.48855	-	-		Weilbull	Model fitting
Log-normal PFS survival model in Nivolumab plus ipilimumab	Meanlog = 1.88660, sdlog = 1.23141	-	-		log-normal	Model fitting
Log-logistic OS survival model in chemotherapy	Shape = 1.7027, scale = 14.1088	-	-		log-logistic	Model fitting
Log-logistic PFS survival model in chemotherapy	Shape = 2.185, scale = 7.392	-	-		log-logistic	Model fitting
**Risk for main adverse events**						
**Nivolumab plus ipilimumab group**						
Diarrhea	0.003		0.0024	0.0036	Beta	Checkmate 743
Increased lipase	0.005		0.004	0.006	Beta	Checkmate 743
Increased amylase	0.003		0.0024	0.0036	Beta	Checkmate 743
**Chemotherapy**						
Anemia	0.36		0.288	0.432	Beta	Checkmate 743
Nausea	0.36		0.288	0.432	Beta	Checkmate 743
Decreased appetite	0.18		0.144	0.216	Beta	Checkmate 743
**Health utility scores**						
Utility of PFS	0.65		0.52	0.78	Beta	(24)
Utility of PD	0.47		0.376	0.564	Beta	(24)
**Cost, $/per cycle**						
Cisplatin	48.6108	38.88864	58.33296	9.72216	Gamma	2022 Payment allowance limits for Medicare
Carboplatin	55.80288	44.6423	66.963456	11.160576	Gamma	2022 Payment allowance limits for Medicare
Pemetrexed	12,832.512	10,266.01	15,399.0144	2,566.5024	Gamma	2022 Payment allowance limits for Medicare
Nivolumab	18,419.94	14,735.95	22,103.928	3,683.988	Gamma	2022 Payment allowance limits for Medicare
Ipilimumab	11,258.31	9,006.648	13,509.972	2,251.662	Gamma	2022 Payment allowance limits for Medicare
Laboratory_test	157.5	126	189	31.5	Gamma	
Follow-up	59.2	47.36	71.04	11.84	Gamma	
Administration	69.81	55.848	83.772	13.962	Gamma	
Best supportive care	117.1	93.68	140.52	23.42	Gamma	(25)
Pembrolizumab	21,479.6	17,183.68	25,775.52	4,295.92	Gamma	2022 Payment allowance limits for Medicare
Vinorelbine	227.2756	181.8205	272.73072	45.45512	Gamma	2022 Payment allowance limits for Medicare
**Expenditures on main AEs, $**						
Diarrhea	303	242	363		Gamma	(26)
Increased lipase	2,933	2,346.4	3,519.6	586.6	Gamma	(27)
Increased amylase	2,933	2,346.4	3,519.6	586.6	Gamma	(27)
Anemia	493.04	246.52	739.56	98.608	Gamma	(28)
Nausea	218.27	174.616	261.924	43.654	Gamma	(29)
Decreased appetite	115.4	103.8	126.9	23.08	Gamma	(30)
**Disutility due to AEs**						
Diarrhea	−0.047	−0.0564	−0.0376	−0.0094	Beta	(31)
Increased lipase	−0.47	−0.564	−0.376	−0.094	Beta	(27)
Increased amylase	−0.47	−0.564	−0.376	−0.094	Beta	(27)
Anemia	−0.09	−0.108	−0.072	−0.018	Beta	(29)
Nausea	−0.048	−0.0576	−0.0384	−0.0096	Beta	(29)
Decreased appetite	−0.038	−0.0456	−0.0304	−0.0076	Beta	(31)
**Risk for Subsequent therapy**						
**Nivolumab plus ipilimumab group**						
Immunotherapy	0.033	0.0264	0.0396	0.0066	Beta	Checkmate 743
Chemotherapy	0.432	0.3456	0.5184	0.0864	Beta	Checkmate 743
**Chemotherapy**						
Immunotherapy	0.202	0.1616	0.2424	0.0404	Beta	Checkmate 743
Chemotherapy	0.315	0.252	0.378	0.063	Beta	Checkmate 743

The calculated drug dose are based on the actual clinical trials. In the Nivolumab plus ipilimumab group, the patients received nivolumab (3 mg/kg intravenously once every 2 weeks) plus ipilimumab (1 mg/kg intravenously once every 6 weeks). In the chemotherapy group, the patients received platinum plus pemetrexed chemotherapy [pemetrexed (500 mg/m^2^ intravenously) plus cisplatin (75 mg/m^2^ intravenously) or carboplatin (area under the concentration-time curve 5 mg/mL per min intravenously)]. We assumed that the average body surface area was 1.68 m^2^ (34). When patients disease progressed, we assumed that all patients who disease progressed had follow-up treatment. It is important to note that the systemic therapy other than PD-(L)1 inhibitors in the subsequent therapies for unresectable MPM, which we chose based on the NCCN 2022.1 guideline (https://www.nccn.org/professionals/physician_gls/pdf/mpm.pdf), is oxaliplatin, leucovorin plus fluorouracil therapy (oxaliplatin 85 mg/m^2^ IV on day 1, Leucovorin 200 mg/m^2^ IV on day 1,2,fluorouracil 1,000 mg/m^2^ IV continuous infusionover 22 h on day 1, 2).

### Sensitivity analyses

A one-way sensitivity analysis was performed to explore the influence of uncertain parameters on the ICER. Each parameter was independently changed by assuming ±20% of the expected value to determine the obvious influence on decision-making. Probabilistic analysis (PSA) was used to randomly sample all the parameters from a specified distribution to further explore the uncertainty and relevance of the model's parameters. According to the parameter type, we selected the appropriate distribution for each uncertain parameter: the cost of the adverse reactions to drugs and treatment is the gamma distribution. The risk of adverse reactions, and the health utility scores including PFS, OS, and AE are the beta distribution. We performed a second-order Monte Carlo simulation of 10,000 iterations and generated a cost-benefit acceptability curve (CEAC) to show that Nivolumab plus ipilimumab is cost-effective with different WTP thresholds.

## Results

### Base-case analysis

The result of base-case analysis about the cost and effectiveness of Nivolumab plus Ipilimumab group and chemotherapy group in patients with unresectable malignant pleural mesothelioma was shown in Table 1. According to our analysis, the incremental cost of Nivolumab plus Ipilimumab ($292,319.48, 1.11 QALYs) vs. chemotherapy ($95,715.26, 0.58 QALYs) is $196,604.22 and the QALYs is 0.53. The ICER values ($371,861.36) are higher than the United States's triple GDP per capita threshold in 2021 ($207,659) (Table 2).

**Table 2:** Base-case analysis results.

**Strategies**	**Cost**	**Incr cost**	**LYs**	**Incr LYs**	**ICER/LYs**	**QALYs**	**Incr QALYs**	**ICER/QALYs**
**All patients group**								
Chemotherapy	95,715.26		0.65			0.58		
Nivolumab plus ipilimumab	292,319.48	196,604.22	1.58	0.92	213,082.80	1.11	0.53	371,861.36

*Incr cost, incremental cost; Lys, life-years; Incr Lys, incremental life-years; QALYs, quality-adjusted life-years; Incr QALYs, incremental quality-adjusted life-years; ICER, incremental cost-effectiveness ratio*.

### Sensitivity analyses

**Figure 1:**
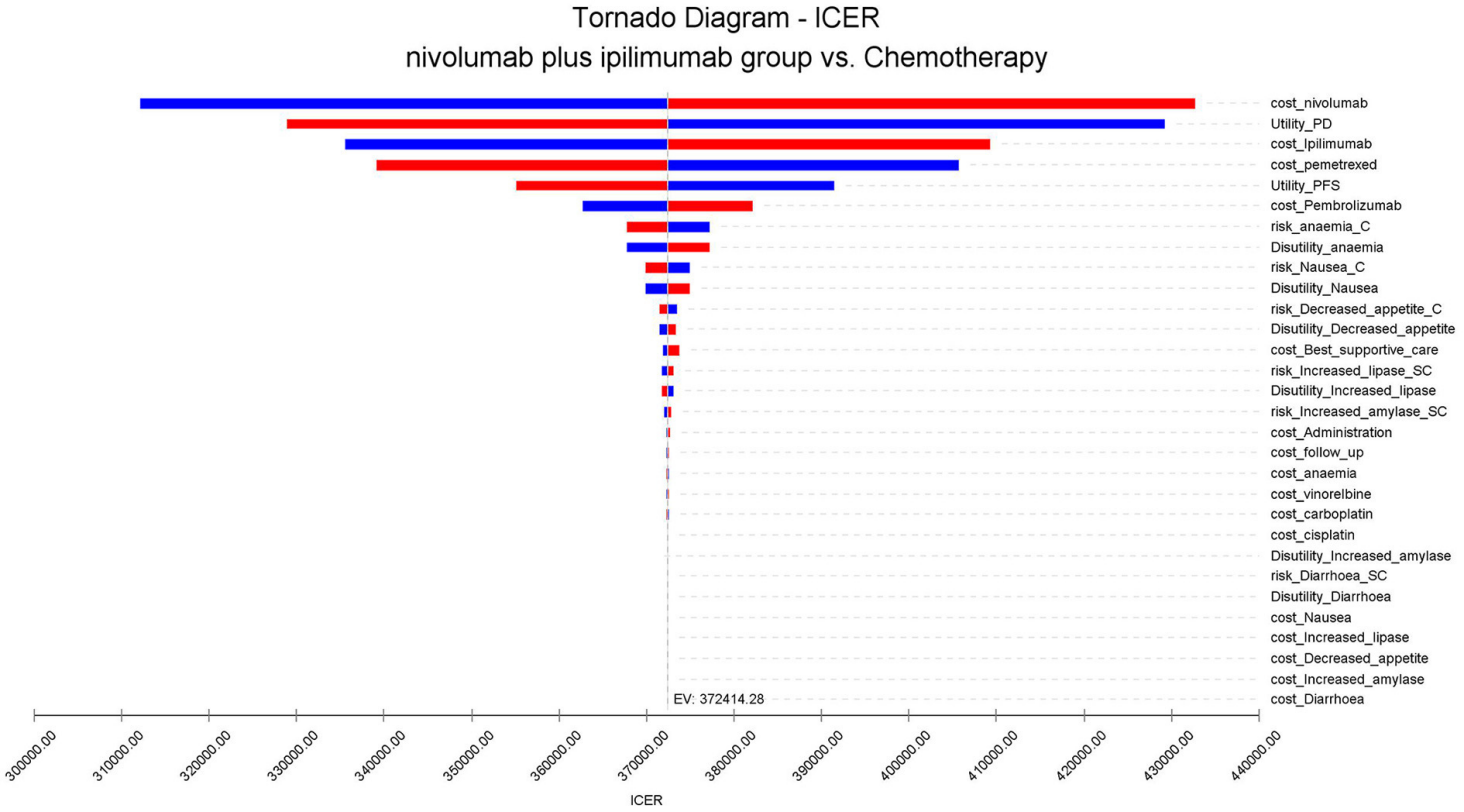
Tornado diagram for one-way sensitivity analysis.

A one-way sensitivity analysis was used to test the robustness of the two population model outputs. Under the condition that the input model parameters change by ±20%, the influence of each parameter on the analysis results is explored. The results are presented in the tornado diagram (Figure 1). The sensitivity analysis results demonstrated that the cost of Nivolumab has the most contributed to it.

### Probability sensitivity analysis

Probability sensitivity analysis (PSA) is applied to test the bias of the multiple model parameters on the analysis results when the multiple model parameters change simultaneously.

**Figure 2:**
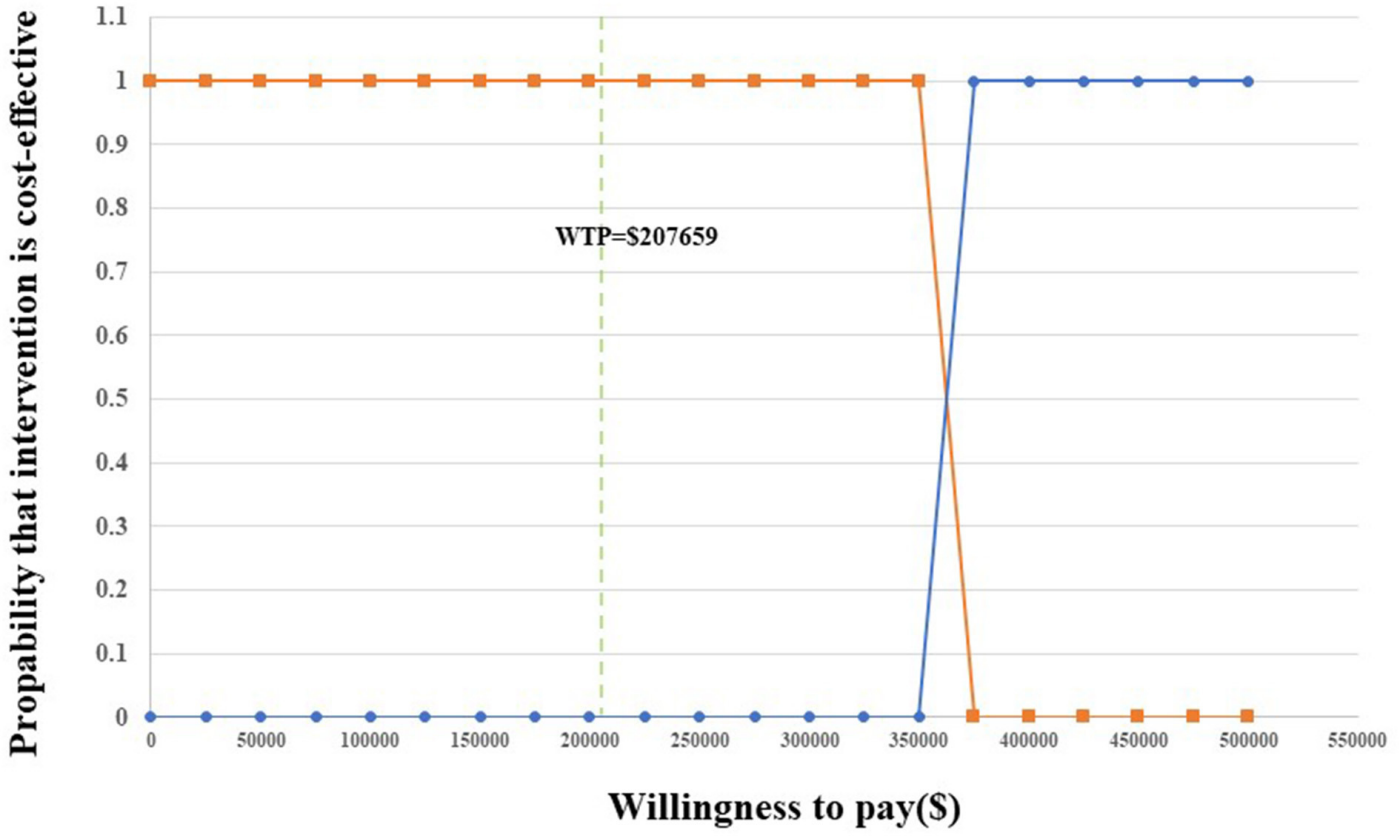
Acceptability curves for the choice of nivolumab plus ipilimumab vs. chemotherapy at different WTP thresholds.

**Figure 3:**
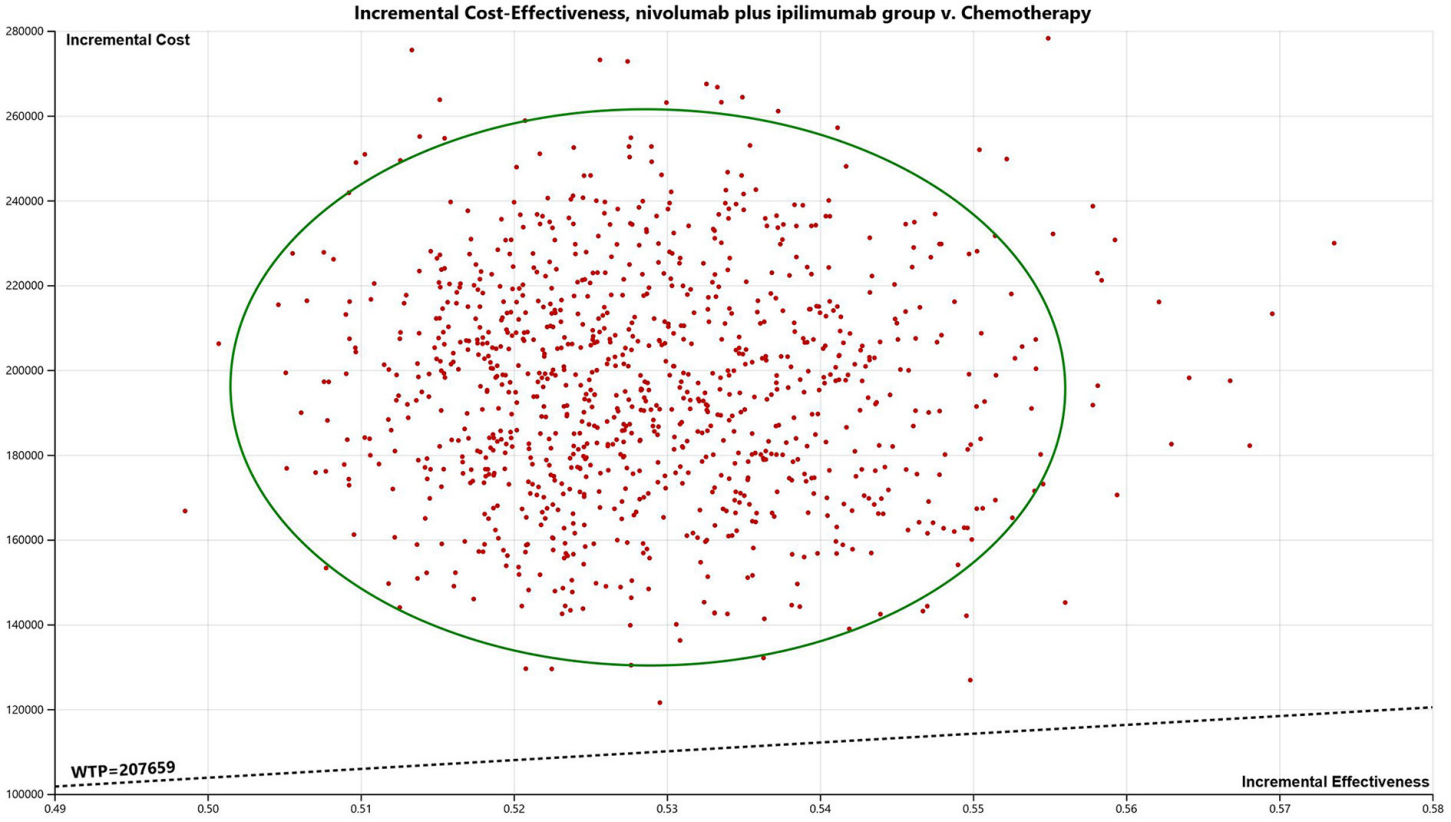
Incremental cost-effectiveness scatter plot (nivolumab plus ipilimumab vs. chemotherapy).

The results are presented through cost-effectiveness acceptability curves (Figure 2) and incremental cost-effectiveness scatterplots (Figure 3). According to the results, we found that the higher the average social willingness to pay, the higher the probability of Nivolumab plus Ipilimumab producing the cost effect. Under the condition of a payment threshold of $207,659 per QALY, Probabilistic sensitivity analysis showed that there was no probability that Nivolumab plus ipilimumab was cost-effective within the fluctuation range of other model parameters in first-line in unresectable malignant pleural mesothelioma.

## Discussion

The phase III randomized trial Checkmate 743 showed a positive result after comparing the efficiency and safety of nivolumab plus ipilimumab and that of chemotherapy as first-line therapy for unresectable MPM. Beforehand, the only one first-line treatment approved by FDA is a platinum-based chemotherapy combined with pemetrexed. Based on Checkmate 743, nivolumab plus ipilimumab has been approved as a first-line therapy for advanced MPM by FDA in October 2020 (35).

There is no doubt that this is a giant leap forward in terms of immunotherapy for MPM. However, the high price has been the spotlight, the financial toxicity will affect the patients' compliance and even leads to termination and abandonment of treatment (36). Especially for the patients with poor ECOG performance status and patients aged 75 years or older on the account of this subgroup didn't benefit from nivolumab plus ipilimumab in current data from Checkmate 743. So, further consideration needs to be given to whether nivolumab plus ipilimumab have a cost-effectiveness advantage as first-line treatment for patients with unresectable MPM.

Based on CheckMate 743 clinical trials and the latest population data and drug prices in USA, our study is the first analysis to evaluate whether Nivolumab plus ipilimumab is more cost-effective than chemotherapy as first-line treatment for treatment-naive advanced MPM with different histology subtypes and PD-L1 expressions from the American payers' perspectives. We obtained the data of Nivolumab plus ipilimumab group and chemotherapy group through the clinical trial CheckMate 743, so we could only conduct cost-effectiveness analysis on these two groups. Our analysis shows that with a WTP threshold of $207,659/QALY for the two groups, the Nivolumab plus ipilimumab group may not be cost-effective to be the first-line for unresectable MPM. We also performed subgroup analyses for all the subgroups mentioned in the clinical trials, unfortunately, the subgroup analysis implied that Nivolumab plus ipilimumab was not a cost-effective strategy across all the patient subgroups. Yet in Checkmate 743, there was a big gap between non-epithelioid [HR 0.46 (95% CI 0.31–0.68)] and epithelioid histology [HR 0.86 (0.69–1.08)] in overall survival of nivolumab plus ipilimumab group and expression level of PD-L1 affects the prognosis of the chemotherapy group, which also called for the further stratified analysis of more heterogeneous epithelioid subtypes.

One-way sensitivity analysis demonstrated that reducing the cost of drugs was the most influential factor to the result of cost-effectiveness. Since the therapeutic effect of the experimental group was better than that of the control group, and chemotherapy was in the control group, the price largely determines the cost-effectiveness. The lower the price of cisplatin, carboplatin, pemetrexed, the lower the cost-effectiveness of the experimental group. There also have been some economic studies on chemotherapy for MPM. A comprehensive review of cost-effectiveness analysis published in 2013 by CHRISTEL C.L.M. BOONS shows that PC (pemetrexed/cisplatin) was not regarded to be cost-effective for the treatment of MPM in the UK. However, the increased cost is considered to be reasonable on account of the lack of efficient treatment (24). After the exploration of targeted therapy for MPM, a study by Mei Zhan on the cost-effectiveness of additional bevacizumab to pemetrexed plus cisplatin in the treatment of unresectable MPM predicted to increase the cost by $81,446.69 with a gain of 0.112 QALYs, which is equal to an ICER of $727,202.589/QALY (37). The study found that additional bevacizumab to PC is not a cost-effective option in China, putting the choice of treatment for advanced MPM into an awkward position to some extent. So, people turn to put focus more on immunotherapy. It has been shown that nivolumab plus ipilimumab is cost-effective for patients suffered from cancer, such as advanced NSCLC with PD-L1 expression level <1%, PD-L1-positive advanced renal-cell carcinoma from the US payer's perspective (34, 36). However, most of the current cost-effectiveness analysis data show that chemotherapy is the more preferred choice than nivolumab plus ipilimumab to treat advanced NSCLC (38). Chemotherapy is much less expensive than immunotherapy in most cases, so it is easier to show cost-effectiveness. Therefore, we conclude that PD-1 inhibitors may be difficult to recommend as cost-effectiveness options for first-line recommendations in unresectable MPM.

We found that when the cost of Nivolumab plus ipilimumab was reduced by 34% (ICER/QALY = 205,679), the ICER was $207,659/QALY which was cost-effective. Therefore, changing the price of Nivolumab plus ipilimumab is an effective feasible strategy to achieve efficient use of them. Medical insurance authority will negotiate with pharmaceutical companies to ensure reasonable drug prices and adjust the medical insurance list to reduce the medical burden on patients.

## Limitations

Our study has some limitations. First, our research is based on Checkmate 743, where there are some inadequacy itself, including the very limited beneficiaries of immunotherapy and the lack of further stratified analysis of epithelioid subtypes (39). Second, CheckMate 743 is a phase three randomized controlled trial, and we used this model to simplify the study. For instance, regarding the adverse reactions, we selected the three to four main AEs that would lead to errors. Third, the study's data originated from the CheckMate 743 trial. Due to the limitation of the number of patients included in the trial, we could not perform a larger-scale analysis, and the trial did not provide the follow-up survival data for patients. We relied on the survival data based on the trial and performed a reasonable extrapolation to predict the long-term survival of patients. This will inevitably be different from the data of real-world patients obtained through regular follow-ups. Fourth, since CheckMate 743 does not disclose the specific health data of patients, our PFS and PD effectiveness were derived from previously published related studies. This may be different from the actual situation of the study. Fifth, we only considered the cost impact and utility reduction caused by the three to four main AEs. The utility reduction caused by specific adverse reactions comes from other published literature, which is in line with the real situation. Sixth, the clinical trial is a multicentred and comprehensive study between different countries and races. The treatment plan of the trial, and especially the follow-up treatment of patients, will be adjusted appropriately according to the specific situation. Therefore, more clinical trials are still required to reduce the study population, follow-up treatment, and other factors that impact the results.

## Conclusion

Overall, from the Americans payers' perspectives, compared with chemotherapy, Nivolumab plus ipilimumab a for the first-line treatment of patients aged 18 years or older who were clinically or pathologically diagnosed with locally advanced or metastatic MPM may be not a cost-effective choice at a WTP threshold of $207,659/QALY.

## Data Availability Statement

The original contributions presented in the study are included in the article/supplementary material, further inquiries can be directed to the corresponding author.

## Ethics Statement

This study was based on a literature review and modeling techniques; this study did not require approval by an Institutional Research Ethics Board.

## Author contributions

Z-mY and ZX had full access to all of the data in the study and takes responsibility for the integrity of the data and the accuracy of the data analysis and concept and design, acquisition, analysis, or interpretation of data. Drafting of the manuscript: Z-QT, Z-mY, and HL. Critical revision of the manuscript for important intellectual content, obtained funding, and supervision: QZ and HL. Statistical analysis: Z-mY, ZX, and Z-QT. Administrative, technical, or material support: Z-QT and HL. All authors contributed to the article and approved the submitted version.

## Funding

This work was funded by the National Natural Science Foundation of China (Grant No. 81900201) and Youth Foundation of Xiangya Hospital (No. 2017Q09) to HL.

## Conflict of interest

The authors declare that the research was conducted in the absence of any commercial or financial relationships that could be construed as a potential conflict of interest.

## Publisher's note

All claims expressed in this article are solely those of the authors and do not necessarily represent those of their affiliated organizations, or those of the publisher, the editors and the reviewers. Any product that may be evaluated in this article, or claim that may be made by its manufacturer, is not guaranteed or endorsed by the publisher.
