# Supplementary material for: Cost-effectiveness of nivolumab plus ipilimumab as first-line treatment for American patients with unresectable malignant pleural mesothelioma
Source: Front Public Health. 2022 Jul 22;10:947375. doi: 10.3389/fpubh.2022.947375 (PMC9354521; doi:10.3389/fpubh.2022.947375)
Supplement: Supplementary file 1 [file Data_Sheet_1.pdf]

Supplementary Material

eFigure1: Markov state transition probability diagram.

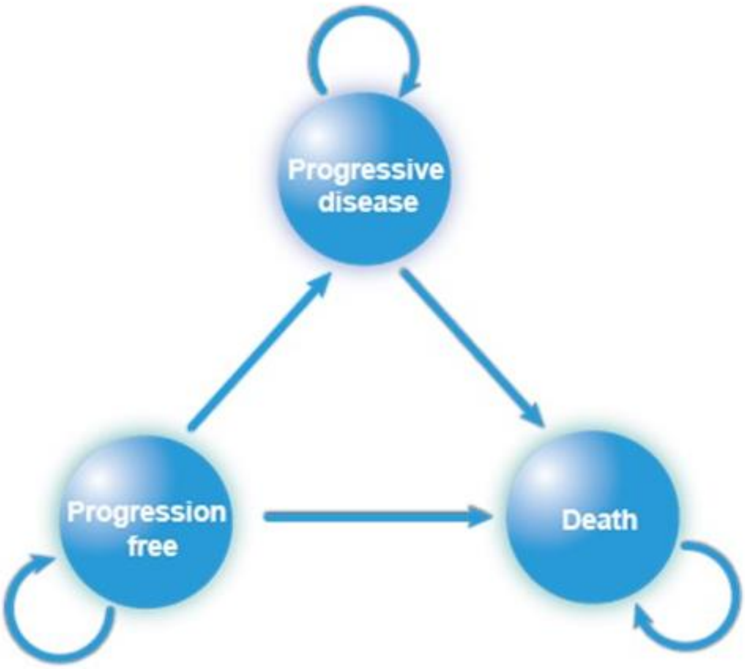

eFigure2: Fitting and extrapolation of Kaplan Meier survival curve for PFS.

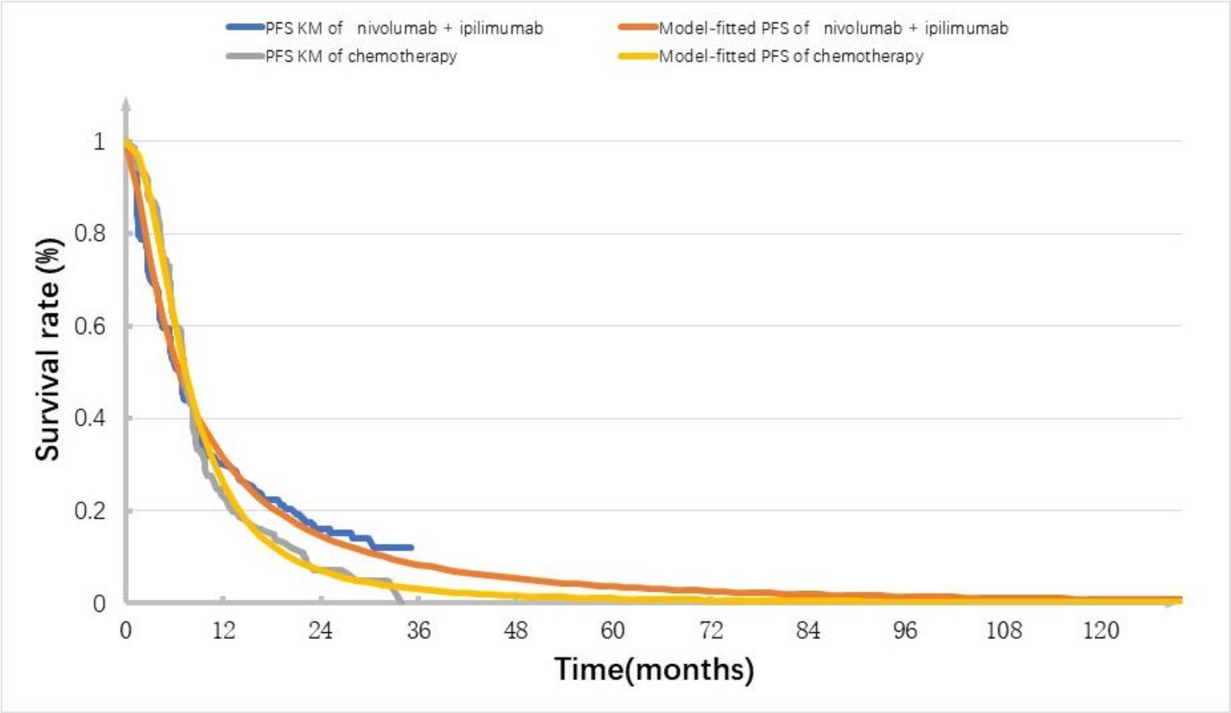

eFigure3. Fitting and extrapolation of Kaplan Meier survival curve for OS.

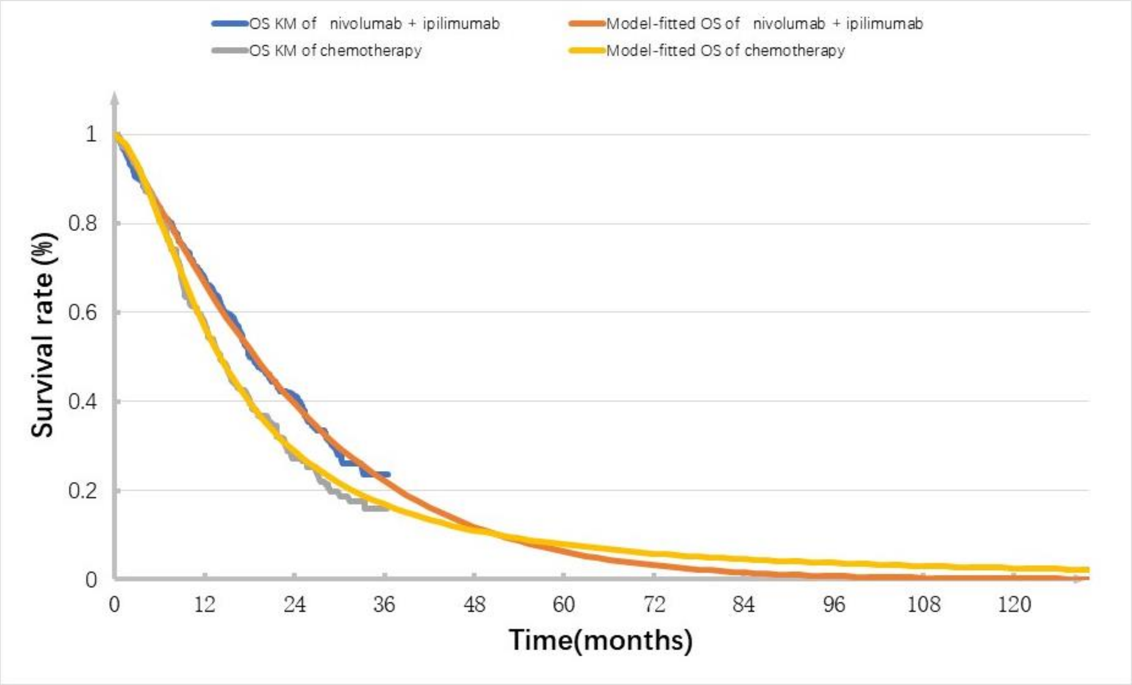

eTable1.AIC and BIC scores of fitted distribution.

Table1.AIC and BIC scores of fitted distribution in all patients from CheckMate 743

Distribution	OS of Nivolumab plus ipilimumab		OS of chemotherapy		PFS of Nivolumab plus ipilimumab		PFS of chemotherapy	
**All patients**	AIC	BIC	AIC	BIC	AIC	BIC	AIC	BIC
Exponential	1710.463	1714.177	1742.953	1746.663	1501.429	1505.143	1422.707	1426.417
Gamma	1705.247	1712.674	1725.998	1733.419	1503.42	1510.847	1378.44	1385.861
Gompertz	1705.421	1712.848	1736.642	1744.062	1488.072	1495.499	1376.032	1387.164
Weibull	1704.805	1712.232	1727.364	1734.785	1502.113	1509.541	1415.815	1423.235
Log-logistic	1712.594	1720.021	1725.553	1732.974	1472.127	1479.554	1388.451	1395.872
Log-normal	1720.415	1727.843	1737.333	1744.754	1466.287	1473.715	1362.945	1370.366

We used Log-logistic,Weibull and Log-normal to fit the OS and PFS curves in our analysis.
Abbreviation: OS=Overall survival; PFS= Progression-free survival; AIC= Akaike information criterion; BIC= Bayesian information criterion.
